# Uterine Displacements and Their Surgical Treatment

**Published:** 1868-06

**Authors:** C. C. F. Gay

**Affiliations:** Buffalo, N. Y.


ART. II.—*Uterine Displacements and their Surgical Treatment.* By C. C. F. Gay, M. D., *Buffalo, N. Y.*

In an article published in the March number of the Journal for 1868, we briefly called attention to the subject of displacements or misplacements of the uterus, venturing therein the opinion that the time was not far distant when dislocations of this organ would most certainly become amenable to surgical treatment or operation, and that the temporizing methods of treatment hitherto employed would give way to the more efficient and radical means afforded by operative interference, and therein cited an instance whereby one of the displacements, viz: procidentia uteri, the treatment of which had heretofore been paliative only, had within a very brief period, by the distinguished labors of Sims and Emmet, become amenable to radical cure by operation.

After briefly alluding in that article to uterine displacements, we promised to resume the subject and speak more fully of them in a future article.

Uterine displacements, then, or to use a term which I like full as well or better, uterine dislocations, the means of diagnosis, and a hint merely at their radical cure by surgical operation, will be the subject of the present paper. The uterus entire may be dislocated in any direction, upwards or downwards, laterally, anteriorly and posteriorly. Three of these dislocations only, for obvious reasons, will demand our present attention, and one of these three misplacements, viz: procidentia uteri, will require only a passing notice until we come to the subject of treatment, for the reason that no new light can be shed upon the subject matter since the cure of this mal-position of the uterus has been so recently brought to perfection by the genius of Dr. Thomas Addis Emmet of the Woman's Hospital, New York.

We are left, then, to treat only of the two remaining essential mal-positions which constitute anti and retro-versions and anti and retro-flexions. Now what do we understand by uterine version? Meigs, writing so far back as 1851, says that retroversion exists when the womb has fallen over backwards, that when the womb tips over in an opposite direction it is antiverted; the former he believes constitutes seventy-five per centum of uterine deviations, and that the retroversion is caused by a distended bladder, and the stretching of the ligamenta rotunda that the fault is in the ligaments and not in the womb. He does not so easily account for the cause of anti-version, but believes this uterine deviation scarcely ever occurs, but when it does occur, is caused not by the *stretching* of the round ligaments as in retroversion, but by their *contraction*. The distended bladder cannot, of course, be brought into requisition as an aid in his explanation of causes.

The definition and causes of version given by this distinguished author are not here produced because they are the best, but for the purpose of contrast between the knowledge of the profession of seventeen years ago and the knowledge possessed by the profession of the present day in relation to the pathology of uterine deviations. The definition is sufficiently correct, but the causes alleged, viz: expansion in the one case and of contraction in the other of the ligamenta rotunda, will not be considered satisfactory to the profession of the present day.

Sims, in his work on uterine surgery, describes anti-version to be that displacement of the womb which is found when the fundus lies just behind the symphysis pubes and when the os is far back towards the hollow of the sacrum. The uterus is retroverted when the fundus falls backward under the promontory of the sacrum, or whenever it passes an angle of forty-five degrees in that direction from its normal position. There are, he says, different degrees of this version.

Anti-version, says Sims, is dependent on a variety of causes, the chief are the shortening of the utero-sacral ligaments and the presence of a fibroid, a small tumor of the body of the uterus posteriorly will produce retroversion, while the same sized tumor of the cervix posteriorly will produce anti-version, and *vice versa*. Granular engorgement of the anterior or posterior lip accompanied by a corresponding engorgement or hypertrophy of the walls of the uterus are included among the causes of these deviations.

Again, sometimes the uterus bends upon its own axis in consequence of an abnormal elongation either of the supra or infravaginal portion of the organ. Sometimes the uterus is undeveloped, entirely too small, and it is not uncommon to find it overdeveloped, with the supra-vaginal portion of the cervix long and slender, and when this is the case the fundus must of necessity fall one way or another, producing anti-version or flexion. The diagnosis of these versions is made easy, and the means of diagnosis are well understood, therefore we need not dwell upon them here.

We desire to call attention to the views of one other authority as corroborative evidence of the rapid change taking place among specialists in uterine diseases and deviations, and also for the purpose of comparison with the views entertained by those who cultivated this special department of female maladies but a few years since.

Dr. Pallen of St. Louis, may be regarded authority, as he is the author of the Prize Essay upon Uterine Abnormities, published in the Transactions of the American Medical Association. As the views enunciated in our former article on "uterine surgery," published in the March number of the Journal for 1868, are in some respects quite similar to those published by Dr. Pallen in his Essay, it would be considered a somewhat suspicious coincidence did we not state that our paper was written two months and published one month before the Prize Essay fell under our observation.

Dr. Pallen, if he does not state it in so many words, at least intimates his belief that versions singly, alone, and uncomplicated with flexion, never exist as a general rule, but if they do happen, they stand as exceptions to the rule, and that the uterine deviations met with are those of flexions and not of versions. I quote him; he says: "Version without flexion in a greater or less degree is rarely met with, in fact such a condition has never existed *absolutely* in any case I have seen, unless it were due to the presence of a fibroid either in or on the body of the uterus, and which caused the misplacement by mere force of gravity."

Whether these views ought to be accepted by the profession as well-founded or not we do not undertake to say, but we do think that there are good and sufficient reasons for the belief that the progress of our knowledge upon the pathology of uterine deviations will soon bring us up to, and in harmony with these views, but that any advance in our knowledge of these uterine deviations will compel us to strike from the present vocabulary the term version, we cannot at present believe; at all events we must accept the fact that the profession is at present in a transition state upon the subject of the so-called versions. It is very true that one cannot readily conceive how version may exist without some degree of flexion, but we have greatly erred if we have not correctly diagnosed version uncomplicated with flexion, and doubtless we have sometimes erred by calling version what should have been named flexion. The position of the os in versions ought to enable any one to decide and diagnose correctly without the uterine sound even. The touch is sufficient to ascertain what direction the os takes, and knowing the direction which the os takes, cannot fail to know the direction of the fundus. We have examined women said to have had uterine version, and have concluded the examination with the positive knowledge that a flexion existed.

I am therefore inclined to believe with Dr. Pallen, that in the future we shall perhaps hear less of versions and more of flexions; indeed recent writers upon uterine abnormities say but little comparatively about versions, while great emphasis is placed upon uterine flexions, and it is for this mal-position of the uterus that most, if not all, operations are made at this time. And this leads us to define uterine flexions, and to state concisely what we understand by the terms anti and retro-flexions.

The best and most recent writers differ somewhat, but not essentially in defining these deviations. Pallen says anti-flexion exists when the body of the uterus is bent forward upon the neck of the uterus, the latter remaining in its normal position, or when the neck is bent forward upon the body of the uterus, the latter remaining in its normal position, or when both body and neck are bent forward; it follows therefore that, when the bending of either body or neck, or both together, is backwards, we have what constitutes retroflexion. The definition here given may not be the most acceptable, but it seems to me as good as any, and shall therefore adopt it as our own.

We think now that we have sufficiently defined the misplacements of the uterus, and made clear the views of some of the most recent writers upon the subject matter to enable any one to have intelligent views in regard to the present state of our knowledge bearing upon the subject of uterine dislocations. We shall, therefore, advance to the subject of diagnosis and causation, and upon this portion of our subject need not occupy much time, for there is little to be said upon the subject of diagnosis of uterine displacements, but the little that need be said is all-sufficient, and may be included under the two heads of the manner and means of diagnosis; of the first, the patient should be placed upon a suitable table or chair, and be instructed to take the left lateral semi-prone position recommended by Sims, and next by means of the Sims' speculum with an assistant, or Emmet's speculum without an assistant, the silver uterine probe and tenaculum, the parts to be examined are readily brought into view and diagnosis accurately made beyond a shadow of doubt or difficulty by bi-manual palpation and the introduction of the sound.

Different views have been advanced by members of the profession touching the causes of anti-flexion and retroflexion. Emmet believes atrophy and fatty degeneration at the seat of flexure to be among the chief causes. Sims, that the flexure is dependent upon many causes, some of which are given under the head of anti-version, and therefore will not be repeated in this place. Other authors, according as they have had different facilities for observations assign different causes, but all agree in locating the primary cause at or upon or within the neck of the uterus. Whatever the primary cause may be which produces the flexure, it is quite easy to comprehend the fact that if a flexure have existed for some considerable time, that the portion of the neck looking toward the flexure must become atrophied, absorbed and its tonicity impaired, while the muscular fibres, of that portion of the neck looking away from the flexure, or in other words the convexity of the flexure, having maintained for some considerable time a partially tense condition, lose at length their contractile power. In a word, the equilibrium existing in a normal state, between the muscular force of either side the neck being destroyed, by whatever cause, cannot now be regained or restored by any inherent power the unimpregnated uterus or the muscular tissue thereof may possess, hence the surgeon's art is evoked to do one of two things—to straighten the cervical canal, an operation which is at present often made with success for the purpose of relieving dismennorrhœa and the sterile condition, still permitting the uterus to remain flexed, or the surgeon's art must be evoked to devise and perform an operation which shall be sufficient to accomplish more than the mere straightening of the canal, but which shall be sufficient to restore the organ to its normal position and its normal relation to other viscera contained within the female pelvis.

Flexures, simply of themselves, I am not aware, are a cause of physical disability when the climacteric period is once passed, and I am quite sure that in many instances flexions may exist for considerable length of time without inconvenience or impairment of health before this period arrives, but in many women flexure is a prolific source of evil of both physical and mental, of local and constitutional derangement. It is believed by some author—I cannot just now say whom, but I think Dr. Emmet—that antiflexions and retroflexions are a cause of phthisis pulmonalis.—Flexions in the main impair the powers of digestion, and during the menstrual excitement, caused by the attendant pain, nervous prostration, and in this way in due process of time may lay the foundation for and lead to the development of tuberculosis, especially should there be any constitutional tendency to such devel opment.

The two misplacements of the uterus known by the names procidentia and flexure, demand our consideration, only, when we come to speak of treatment, especially surgical treatment, for these two deviations have latterly occupied the profession more, and greater progress has been made in the knowledge of the pathology and treatment of these abnormities than in any and all other deviations to which the uterus is liable. The treatment of procidentia uteri is palliative and radical. The first, as all know, consists in reducing, so to speak, the dislocation of the womb, and when reduced to maintain it in its normal position by the use of a pessary. The use of astringent washes is recommended, for astringents tend to diminish the vaginal calibre and to prevent the inversion of the vagina. More or less vaginal inversion takes place whenever the os protrudes beyond the vulva into the external world.

For the purpose of retaining the os in its normal position within the vaginal canal, a pessary made of gutta-percha tubing from one-quarter to one-half inch in diameter is as good as any. When bent in the form of a ring the ends of the gutta-percha may be secured by means of cork. A pessary thus made will possess the merits of cheapness, if no other, but thanks to the distinguished services of Dr. Thomas Addis Emmet, women are not now obliged to carry through life a pessary within the vagina. The operation which he devised, and has so many times successfully performed, is so simple in itself, so painless in its performance, and attended with so little danger, that we marvel that any one, unless ignorant of the benefits to be derived from the operation, would hesitate for one moment to submit to the radical cure.

As, perchance, some of our readers may not know the character of the operation, we will state in genernl terms and in the use of the fewest possible words of what it consists, without describing the operation in detail. The woman is placed upon the table in the left lateral semi-prone position of Sims, the tips of the fingers of both hands are placed against the protruding os, and as the os is crowded upward within the vagina, folds of mucous membrane will enclose it and help keep it in situ when restored to its proper position. Emmet then produces anti-flexion of the womb by means of bi-manual palpation. For this step in the process of the operation Emmet claims originality, and also claims that the anti-flexion facilitates the operation greatly. Then, with the curved scissors the mucous membrane, nearly one-half inch in width, is denuded, beginning at a point near the meatus urinarius, and running up to either side the os by two divergent lines, the termini of the two divergent denuded surfaces may now be two or even three inches apart, and are connected, by denuding the mucous membrane transversely just below the os, and when comp'ete will leave a triangular space untouched by the scissors; these opposing raw surfaces are brought together by fifteen or more silver sutures secured by twisting over the wire-holder, when the operation is completed without the use of chloroform or pain — When the parts have healed the os will have gocd firm support by partia'ly resting upon a floor of mucous membrane and by reason of the diminished calibre of the vaginal canal.

This operation, which is of recent date, is one of the most important and successful of any in the annals of modern surgery. We wish we could say as much for operative interference in flexions. The treatment of flexion is at present only palliative; we wish we could say it was radical and curative. The time is not far distant, we apprehend, when we may be able to set up claims for the radical treatment of flexions, treatment we mean by operation; this certainly is not too much to expect or to say when we call to mind the present increased facilities and resources of the profession, and the greater activity of original and independent thinkers at present, prosecuting studies in the science of gynaecology.

It would necessitate the writing of a hundred pages or more, instead of a single page, should we attempt to give the present state of our knowledge of all the various means which have been and are now used, or to indicate our own views upon the subject matter of the palliative treatment of flexions. We should not know where to begin or where to leave off; to properly write up the medical literature of this branch of our subject would require of us to describe pessaries of divers forms and patterns, and to accompany our description with diagrams until a book might be filled from preface to fly-leaf with them, and this done no good would accrue to any one from the task.

Operations upon the uterus, or, to be more specific, operations upon the os or cervix for the mal-position called flexion, are at present limited in their use to straighten the canal for the purpose of preventing dismenorrhœa and relieving the sterile condition, but I do not understand that this operation relieves the flexure proper, for that remains, though the cervical canal be made straight. I am not unconscious, however, that the operation is called an operation for the rectification of flexure. We want an operation which shall supercede the necessity of the performance of an operation for the mere and sole purpose of rendering the cervical canal straight, and we believe as we before indicated, that such operation is almost within the grasp of the profession. We have recently made some experiments with this object in view, but have been too limited in our opportunities to accomplish any thing tangible or worthy of record. We are at present treating a retroflected womb, and can easily replace the fundus in its normal position with the delicate silver probe or Sims' sound, but the moment the sound is withdrawn over goes the uterus again; if now we seize with the tenaculum the anterior lip and make slight traction, and then reposit the womb with the sound we are able to hold the womb in position with our tenaculum. It does not require much acumen to see that if the womb could thus be long enough supported to enable the muscles, so long tense and stretched upon the cervix anteriorly, and the muscles which have become atrophied upon the cervix posteriorly, to regain their tonicity, strength and contractile power, that if the equilibrium of the muscular power anteriorly and posteriorly, which has been long lost, could now be regained, that the womb would as certainly remain in its normal position as that it ever occupied such position.

The question arising, then, in the mind of any original thinker would be this: might it not be possible by denuding the cervix near its junction with the vagina, or infra-vaginal portiou of the cervix of sufficient amount of mucous membrane to enable us to obtain the requisite amount of traction for holding the uterus in place? In retroflexion denude upon the cervix anteriorly, and *vice versa;* double the tissue upon itself and secure the parts in apposition by silver suture, and possibly the cicatrical contraction would be sufficient, after the womb has been reposited by the probe, to hold it in situ. An operation of this character would have the merit of simplicity, if nothing more, but need not be ignored on that account, for if an operation is ever to be devised for the restoration of a flexed uterus, it must of necessity be a simple one.

In speaking of this operation we do not know that we have been clear in trying to convey to others our ideas; probably we may have succeeded in making ourselves well *mis*understood. We are constantly embarrassed with the thought that we are making our paper too prolix, and are ever striving to be brief. Thus have we striven to the best of our ability to fulfill our promise made in the article upon "uterine surgery."

We hope it may not be considered an indelicacy in us to state in this connection that since our former paper was published, we have been gratified to know that the views therein enunciated have received the sanction of such authority as Emmet, and Bozman and Pallen. We never claim perfection for any paper emanating from our pen, but if anything were wanting to add to the sanction of the authority of such names as are above mentioned, to show that the paper alluded to possessed some degree of merit, it could be found in the fact that the paper covering eight pages of the Journal had called forth a so-called review, covering nearly *seven*

*pages* of the Journal—space sufficient, one would think, for the review of a goodly sized book. We must, therefore, accept as a high compliment, the generous attentions paid us, and tender sincerest thanks to our reviewers, individually and collectively, in the abstract and concrete.

